# Supplementary material for: The interactome of intact mitochondria by cross-linking mass spectrometry provides evidence for coexisting respiratory supercomplexes
Source: Mol Cell Proteomics. 2017 Dec 8;17(2):216–32. doi: 10.1074/mcp.RA117.000470 (PMC5795388; doi:10.1074/mcp.RA117.000470)
Supplement: Supplemental Data [file supp_RA117.000470_133922_0_supp_23978_fzffwf.zip › spectra_annotation/mito_DR_spectra_annotation/393-1-14-1-26-1.pdf]

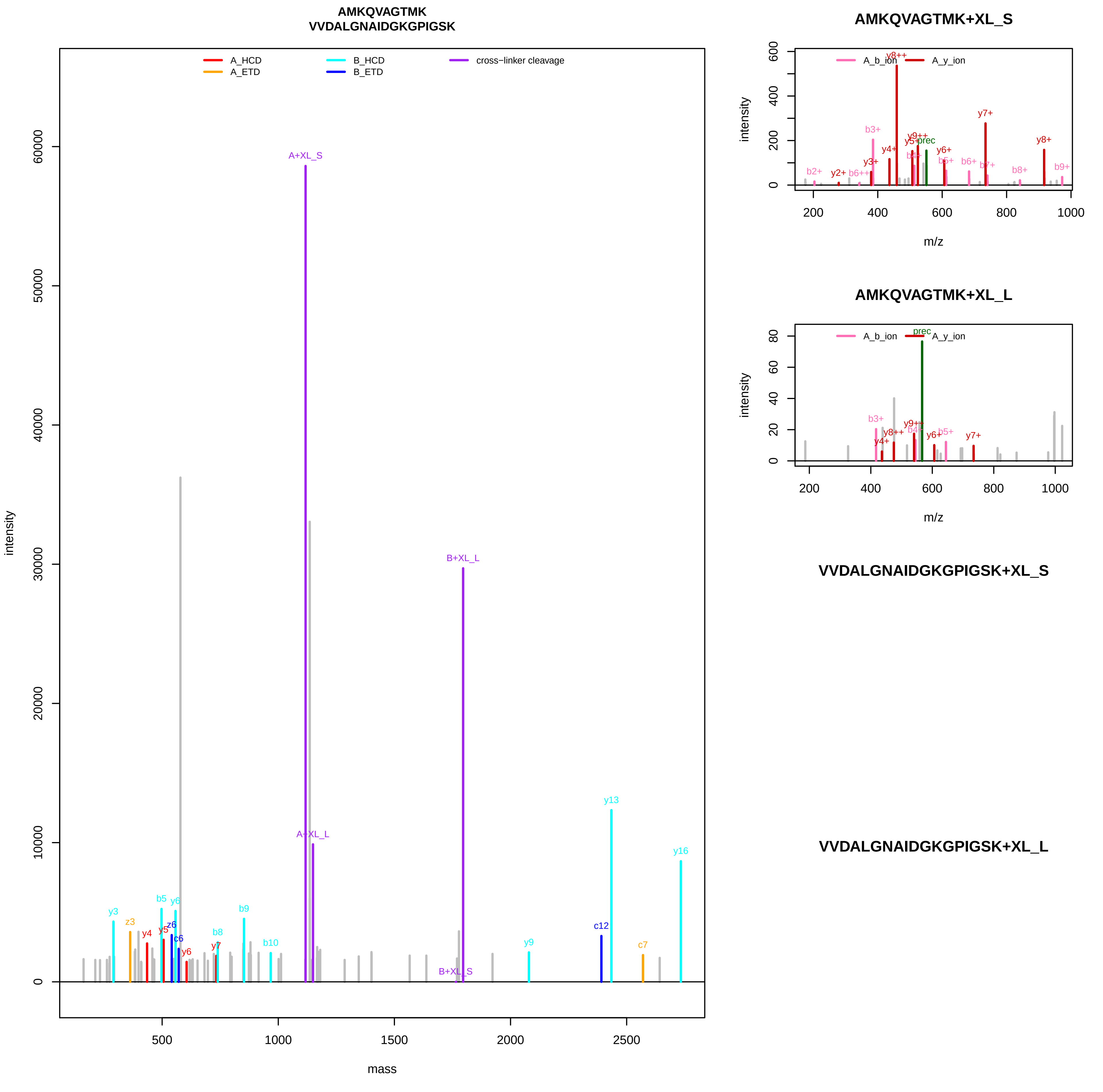
AMKQVAGTMK
VVDALGNAIDGKGPIGSK
A_HCD
A_ETD
B_HCD
B_ETD
cross−linker cleavage
A+XL_S
B+XL_L
A+XL_L
B+XL_S
y3
z3
y4
b5
y5
y6
z6
c6
y6
y7
b8
b9
b10
y9
c12
y13
c7
y16
intensity
mass
0
10000
20000
30000
40000
50000
60000
500
1000
1500
2000
2500
AMKQVAGTMK+XL_S
A_b_ion
A_y_ion
b2+
y2+
b6++
y3+
b3+
y4+
y8++
y9++
y5+
prec
b4+
y6+
b5+
b6+
b7+
y7+
b8+
y8+
b9+
intensity
m/z
0
200
400
600
800
1000
AMKQVAGTMK+XL_L
A_b_ion
A_y_ion
prec
b3+
y8++
y4+
y9++
b4+
y6+
b5+
y7+
intensity
m/z
0
20
40
60
80
200
400
600
800
1000
VVDALGNAIDGKGPIGSK+XL_S
VVDALGNAIDGKGPIGSK+XL_L
